# QSAR and Molecular Docking Studies of Pyrimidine-Coumarin-Triazole Conjugates as Prospective Anti-Breast Cancer Agents

**DOI:** 10.3390/molecules27061845

**Published:** 2022-03-11

**Authors:** Arun Kumar Subramani, Amuthalakshmi Sivaperuman, Ramalakshmi Natarajan, Richie R. Bhandare, Afzal B. Shaik

**Affiliations:** 1 Sathyabhama Institute of Science and Technology, School of Pharmacy, Chennai 600119, Tamil Nadu, India; arunpharma73@gmail.com; 2 Department of Pharmaceutical Chemistry, C. L. BaidMetha College of Pharmacy, Chennai 600097, Tamil Nadu, India; ramalakshmi.arunkumar@gmail.com; 3 Department of Pharmaceutical Sciences, College of Pharmacy & Health Science, Ajman University, Ajman P.O. Box 346, United Arab Emirates; 4 Center of Medical and Bio-Allied Health Sciences Research, Ajman University, Ajman P.O. Box 346, United Arab Emirates; 5 Department of Pharmaceutical Chemistry, Vignan Pharmacy College, Vadlamudi 522213, Andhra Pradesh, India

**Keywords:** coumarin, QSARINS, docking, DHFR, tubulin

## Abstract

Cancer is a life-threatening disease and is the second leading cause of death worldwide. Although many drugs are available for the treatment of cancer, survival outcomes are very low. Hence, rapid development of newer anticancer agents is a prime focus of the medicinal chemistry community. Since the recent past, computational methods have been extensively employed for accelerating the drug discovery process. In view of this, in the present study we performed 2D-QSAR (Quantitative Structure-Activity Relationship) analysis of a series of compounds reported with potential anticancer activity against breast cancer cell line MCF7 using QSARINS software. The best four models exhibited a *r*^2^ value of 0.99. From the generated QSAR equations, a series of pyrimidine-coumarin-triazole conjugates were designed and their MCF7 cell inhibitory activities were predicted using the QSAR equations. Furthermore, molecular docking studies were carried out for the designed compounds using AutoDock Vina against dihydrofolate reductase (DHFR), colchicine and vinblastine binding sites of tubulin, the key enzyme targets in breast cancer. The most active compounds identified through these computational studies will be useful for synthesizing and testing them as prospective novel anti-breast cancer agents.

## 1. Introduction

Cancer is the second leading cause of death worldwide [1]. For decades, conventional cytotoxic chemotherapy has been a key component of advanced cancer treatment in the cancer therapeutic arsenal [2]. However, only a minor improvement in survival rates has been achieved. Recent anticancer drug development is heavily reliant on drug targets, such as proteins, enzymes and receptors, and mechanism-based drug discovery would considerably accelerate the process. [3,4,5]. Various targets reported for anti-cancer activities include ribonucleotide reductase [6], estrogen receptors (ERs) [7,8], aromatase enzymes [9], type I and type II topoisomerases [10], microtubules [11] and dihydrofolate reductase, among others. Although many targets are well-known and validated, still they offer various opportunities.

Dihydrofolate reductase (DHFR) and tubulin proteins of microtubules are of great interest to medicinal chemists, since the inhibition of these sites is an important action of the marketed anticancer drugs methotrexate and vinca alkaloids. The inhibition of DHFR is imparted by compounds with antibacterial [12,13,14], antimalarial [15,16], antifungal [17] and anticancer effects [18,19]. Further, DHFR is an excellent template for enzyme selectivity and antiproliferative effects of antifolates for cancer chemotherapy [20]. DHFR enzyme converts dihydrofolate to tetrahydrofolate by means of NADPH in microbial and eukaryotic cells [21]. Accordingly, it is tangled in the combination of crude material for cell expansion, in both prokaryotic and eukaryotic cells. Trimethoprim (TMP), (2,4-diamino-5-(3′,4′,5′-trimethoxybenzyl) pyrimidine) is the renowned dihydrofolate reductase inhibitor used in urinary tract infections [22,23,24]. Tubulin is an important protein involved in cell division. It contains the α- and β- families, which polymerize to produce microtubules during cell division. These subunits are highly conserved and ubiquitous in eukaryotic cells. Microtubules are manipulated into separate daughter chromosomes during mitosis by rapid construction and disassembly. They are crucial for cellular replication. Furthermore, microtubules support cell morphology and material transport. The interference of microtubules causes cell death by apoptosis. Hence, the mitotic microtubules are novel platforms for cancer chemotherapy. Anticancer drugs halt cell division during mitosis, slowing cell proliferation by inhibiting or promoting tubulin polymerization. Vinca alkaloids such as vincristine and vinblastine, in addition to colchicine and paclitaxel, are well-known tubulin-interactive anticancer drugs [25,26,27,28].

The major challenges with the drugs acting on single targets are drug resistance and innumerable side effects [29,30]. Molecular hybridization technique maces the active pharmacophores with a linker that could simultaneously address more than one sole target. This is a very useful approach for the design of novel drugs against a complex disease such as cancer. In drug development, one of the rational and successful methods is the quantitative structure-activity relationship, which is a crucial step in the development and optimization of lead compounds, and consequently to improving their biological activity. Natural products bind with biomolecular drug targets more readily, as they are the metabolites of living organisms. Thus, they serve as an ideal resource for drug development. It is obvious to choose coumarin, a widely distributed secondary metabolite in plant kingdom and pyrimidine, a common component of nucleic acids, for the present study. Previously, Mohit Sandhuja et al. reported the anticancer activity for uracil–coumarin-based bifunctional molecular hybrids connected with 1,2,3-triazole moiety [31]. By considering the above facts and the report by Sandhuja et al., in the present investigation we utilized QSAR and conducted a detailed QSAR study using “QSARINS” software [32,33,34] for accelerating the drug discovery process for the identification of novel lead compounds with anti-breast cancer activity.

## 2. Results

### 2.1. D-QSAR

#### 2.1.1. Development of Model and Descriptors

Mohit Sandhuja et al. synthesized a series of uracil–coumarin conjugates and reported their anticancer activity [31]. This series was selected for carrying out 2D-QSAR studies in the present study. Table 1 indicates the compounds synthesized by Mohit Sandhuja. The anticancer activity was reported in IC_50_ by Mohit Sandhuja. They were converted to pIC_50_ for carrying out 2D-QSAR studies and included in Table 1. 

#### 2.1.2. Generation of 2D-QSAR Models

For practical use of the QSAR model, robustness (cross validated performance) and predictability (external predictive capacity) are the parameters that determine the superiority of the QSAR model. Four models were selected for QSAR study.

##### Model 1

pIC_50_ = −4.3799 + 0.8065(MAXDP) + 14.2488(SIC0) + 35.9491(JGI7) (1)

ntr = 18, npred = 10, R^2^ = 0.9700, R^2^adj = 0.9635, R^2^−R^2^ adj = 0.0064, LOF = 0.0060, RMSEtr = 0.0515, MAEtr = 0.0422, RSStr = 0.0478, CCCtr = 0.9848, s = 0.0584, F = 150.7739, Q2 LOO = 0.9495, Q^2^LMO = 0.9449, R^2^Yscr = 0.1763, Q^2^Yscr = −0.3864, RMSEcv = 0.0668, MAEcv = 0.0549, PRESScv = 0.0804, CCCcv = 0.6865, R^2^ext = 0.0983, MAEext = 0.0856, PRESSext = 0.0966, RMSEext = 0.0983, CCCext = 0.6865, Q2 F1 =0.4530, Q^2^ F2 =0.4375, Q^3^ F3 = 0.8908 (Equation (1)).

##### Model 2

pIC_50_ = 17.6885 – 0.0831 (AATS7s) − 3.6745 (MATS2c) − 8.2998 (SpMin3_Bhi) + 0.2438(MDEC-33) (2)

Ntr = 20, npred = 6, R^2^ = 0. 0.9883, R^2^ adj = 0.9851, R^2^−R^2^ adj = 0.0031 LOF = 0.0016, RMSEtr = 0.0236, MAEtr = 0.0192, RSStr = 0.0112, CCCtr = 0.9941, s = 0.0273, F = 315.6856, Q^2^ LOO = 0.9796, Q^2^LMO = 0.9740, R^2^Yscr = 0.2090, Q^2^Yscr = −0.4425, RMSEcv = 0.0312, MAEcv = 0.0256, PRESScv = 0.0194, CCCcv = 0.9897, R^2^ext = 0.9726, MAEext = 0.0448, PRESSext = 0.0121, RMSEext = 0.0450, CCCext = 0.9754, Q^2^ F1 = 0.9698, Q^2^ F2 = 0.432, Q^3^ F3 = 0.9575 (Equation (2)). 

##### Model 3

pIC_50_ = 18.2893 − 3.1558 (MATS2c) − 8.6164 (SpMin3_Bhi) − 0.3147 (ETA_EtaP_F) + 0.2471 (MDEC-33) (3)

ntr = 20, npred = 6, R2 = 0.9875, R^2^adj = 0.9842, R^2^–R^2^ adj = 0.0033, LOF = 0.0016.

RMSE tr = 0.0244 MAE tr = 0.0198 RSS tr = 0.0119 CCC tr = 0.9937s = 0.0281F = 296.9651 Q^2^loo = 0.9789 RMSE cv = 0.0316 MAE cv 0.0260 PRESS cv = 0.0200 CCC cv = 0.9894 Q^2^LMO = 0.9738 R^2^Yscr = 0.2095 Q^2^Yscr = −0.4288 RMSE ext = 0.0527 MAE ext = 0.0512 PRESS ext = 0.0167 R^2^ext = 0.9665 Q^2^-F1 = 0.9586 Q^2^-F2 = 0.9220 Q^2^-F3 = 0.9416CCC ext: 0.9677 (Equation (3)).

##### Model 4

MATS2c and ETA_EtaP_F were included in equation 3 along with MATS2c, SpMin3_Bhi MDEC-33. MATS2c and ETA_EtaP_F and contributed negatively to the activity.

pIC_50_ = 20.1355 − 2.8197 (MATS2c) − 9.3329 (SpMin3_Bhi) − 0.3066 (IC1) + 0.2710 (MDEC-33) (4)

R^2^ = 0.9874, R^2^adj = 0.9840, R^2^-R^2^adj = 0.0034, LOF = 0.0017, RMSE tr = 0.0245, MAEtr = 0.0199, RSS tr = 0.0120, CCC tr = 0.9937, s = 0.0283, F = 293.8200, Q^2^loo = 0.9777, R^2^-Q^2^loo: 0.0097, RMSE cv = 0.0326, MAE cv = 0.0270, PRESS cv = 0.0212, CCC cv = 0.9888.

Q^2^LMO = 0.9733, R^2^Yscr = 0.2098, Q^2^Yscr = −0.4428, RMSE ext = 0.0488, MAE ext = 0.0433 PRESS ext = 0.0143, R^2^ext = 0.9703, Q^2^-F^1^ = 0.9644, Q^2^-F^2^ = 0.9330, Q^2^-F^3^ = 0.9499 CCC ext = 0.9720 (Equation (4)).

IC1 parameter is newly included in model 4 and it had a negative contribution to the activity.

#### 2.1.3. Validation of 2D-QSAR Models

The correlation matrix for the best model (model 2) obtained is provided in Table 2.

**Figure 1:**
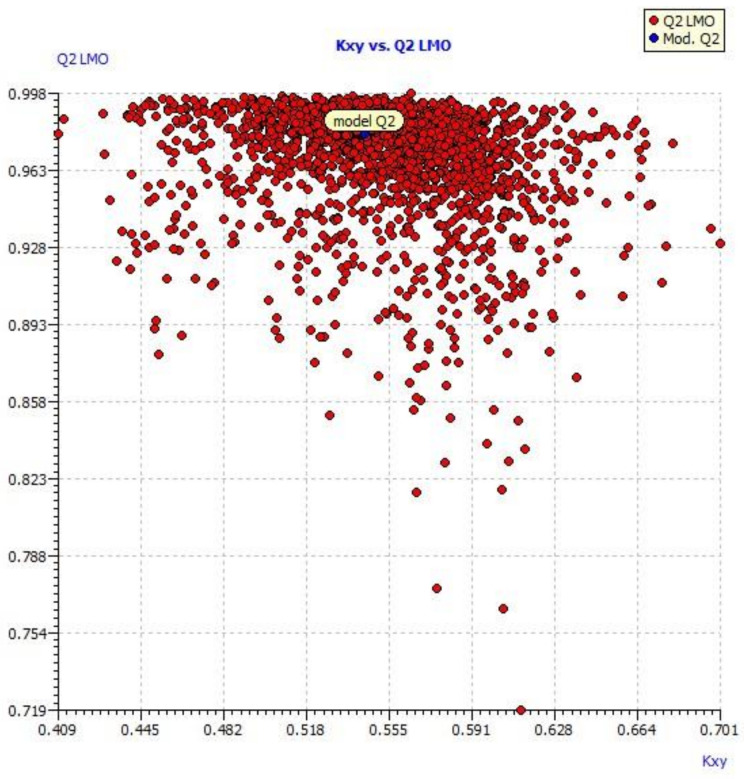
Scatter plot of data set compounds obtained from experimental values and the final model equation.

**Figure 2:**
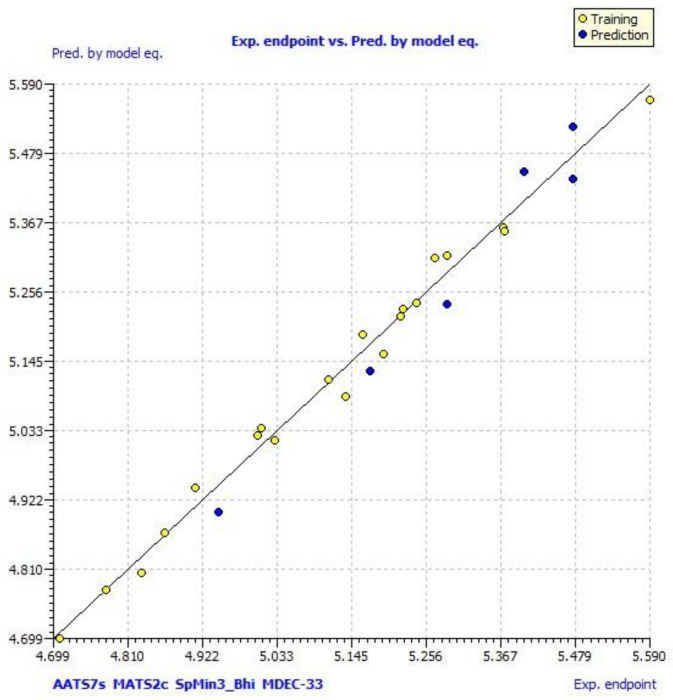
The LMO scatter plot (plot of Kxy vs. Q2 LMO).

**Figure 3:**
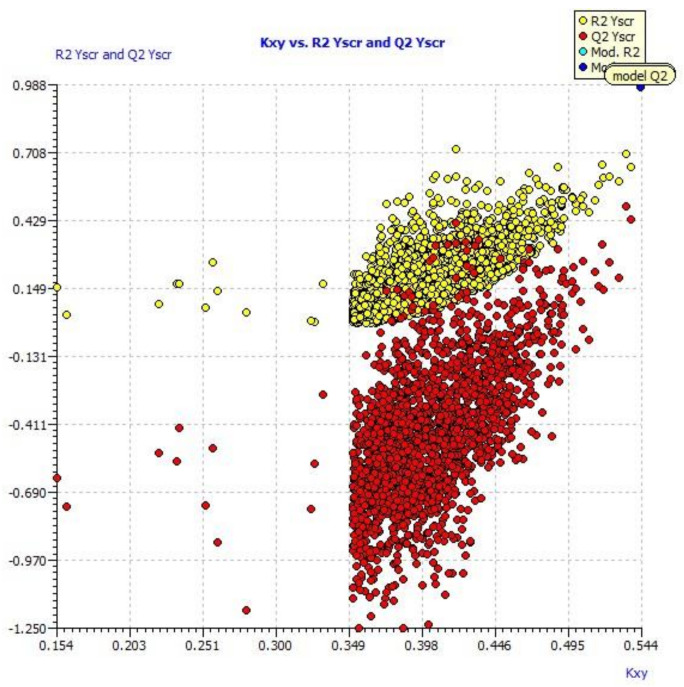
Y-scramble plot (plot of Kxy vs. R2 and Q2 LOO from Y-scrambling procedure).

A scatter plot of data set compounds obtained from experimental values and the final model equation is presented in Figure 1. Figure 2 represents the LMO scatter plot. The Y-scramble plot of Kxy versus R2 Yscr and Q2 Yscr is shown in Figure 3, which shows that correlation coefficients of the final model are much higher than those after endpoint scrambling, and a broken relationship can be evidenced between structure and responses.

pIC_50_ = 18.2893 − 3.1558 (MATS2c) − 8.6164 (SpMin3_Bhi) − 0.3147 (ETA_EtaP_F) + 0.2471 (MDEC-33)

**Figure 4:**
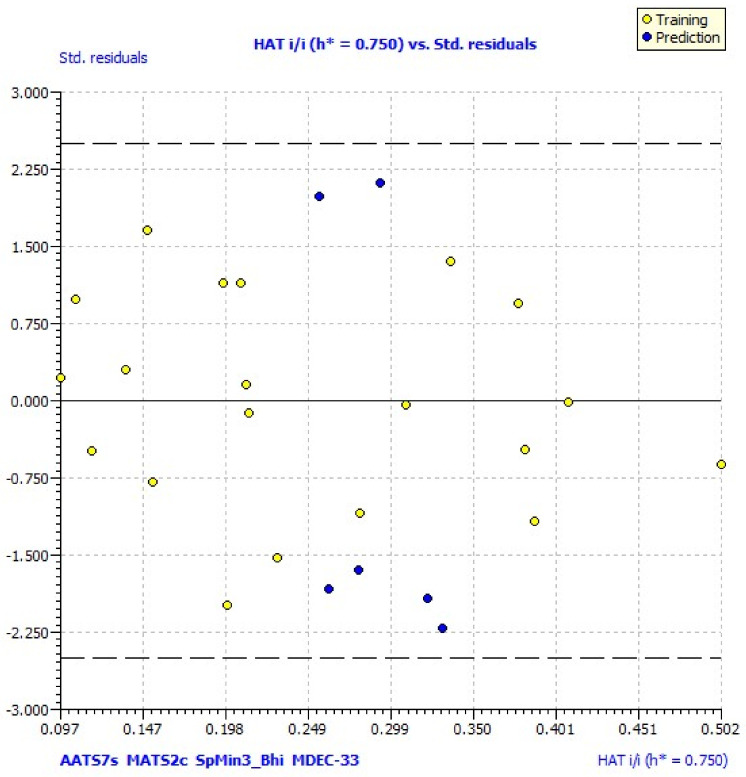
William’s plot of the best model. The dashed lines are the cut-off 3σ and the warning value of HAT (h*, 0.750).

The applicability domain of the model was explained by William’s plot, standardized residuals versus leverage values shown in Figure 4, and it illustrates the prediction and expression. William’s plot indicated that all the molecules are located in the applicability domain of the model, with leverage values lower than the warning h* of 0.750.

From the four models, model 2 was found to be the best equation. The predicted activities calculated from the best model were found to be closest to observed activities (Table 3). Twelve new compounds were designed using ChemSketch software. The novelty of the compounds was confirmed by Scifinder search (Table 4).

Physicochemical properties of designed compounds were predicted using the PaDEL descriptor. Their anticancer activity against MCF7 cells was predicted using the generated QSAR models. 

The novel designed compounds exhibited very high inhibitory activity compared to the most active compound of the original data set. The most active compound in the original data set had an IC_50_ value of 1.55. Its pIC_50_ value was found to be 5.809. All the predicted compounds exhibited pIC_50_ values of more than 9.0 except one compound which had a pIC_50_ value of 8.007. The predicted activities against MCF7 cells using the generated QSAR equation for the compounds **g**, **h**, **i** and **j** were found to be 7.9, 7.80, 8.59 and 8.33, respectively (Table 5).

### 2.2. Docking Studies

**Figure 5:**
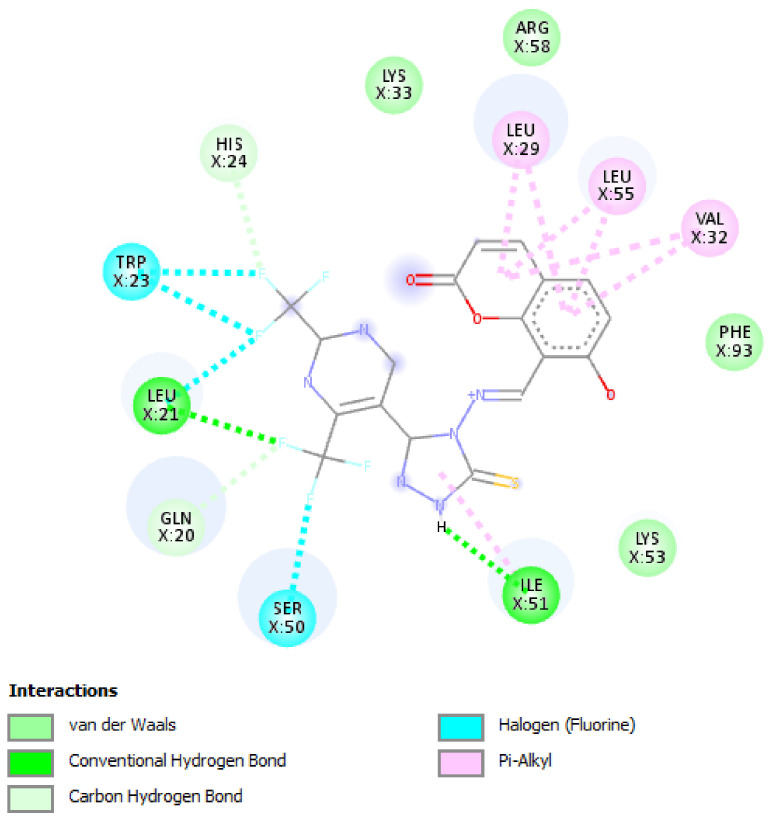
Docking interactions of designed compound **g** with DHFR enzyme.

**Figure 6:**
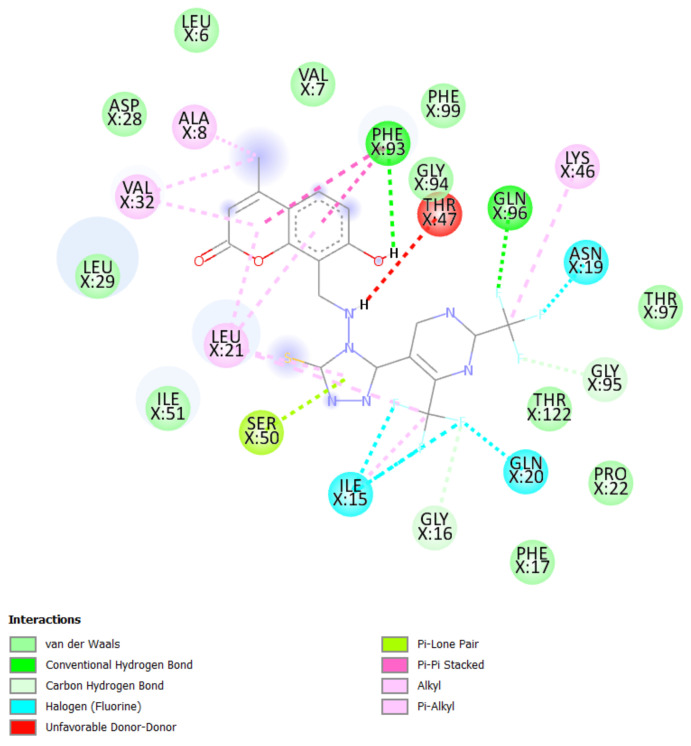
Docking interactions of designed compound **h** with DHFR enzyme.

**Figure 7:**
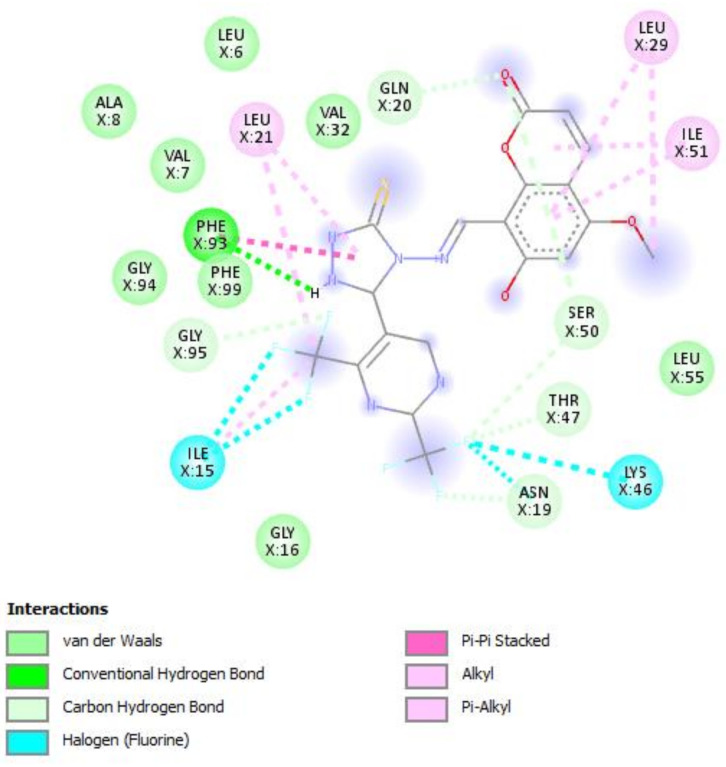
Docking interactions of designed compound **i** with DHFR enzyme.

**Figure 8:**
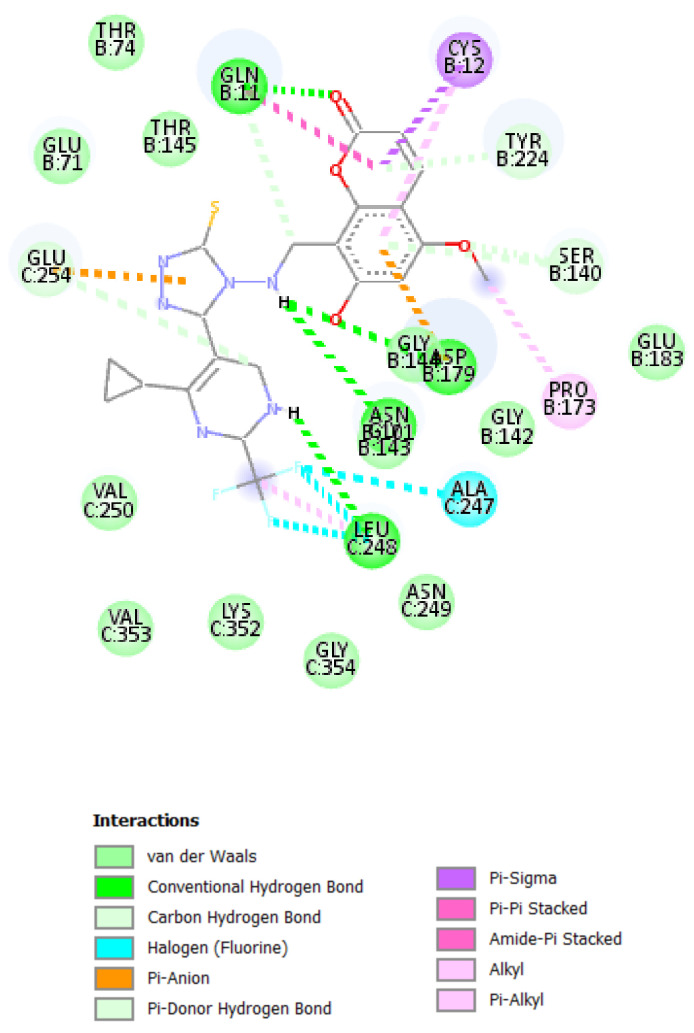
Docking interactions of designed compound **k** with colchicine binding site of tubulin enzyme.

**Figure 9:**
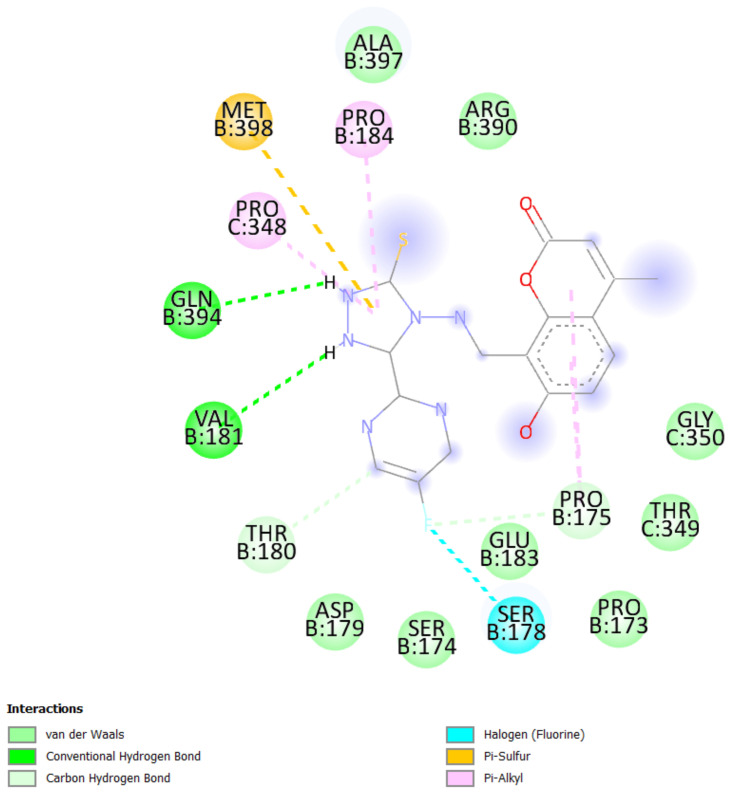
Docking interactions of designed compound **b** with vinblastine binding site of tubulin enzyme.

**Figure 10:**
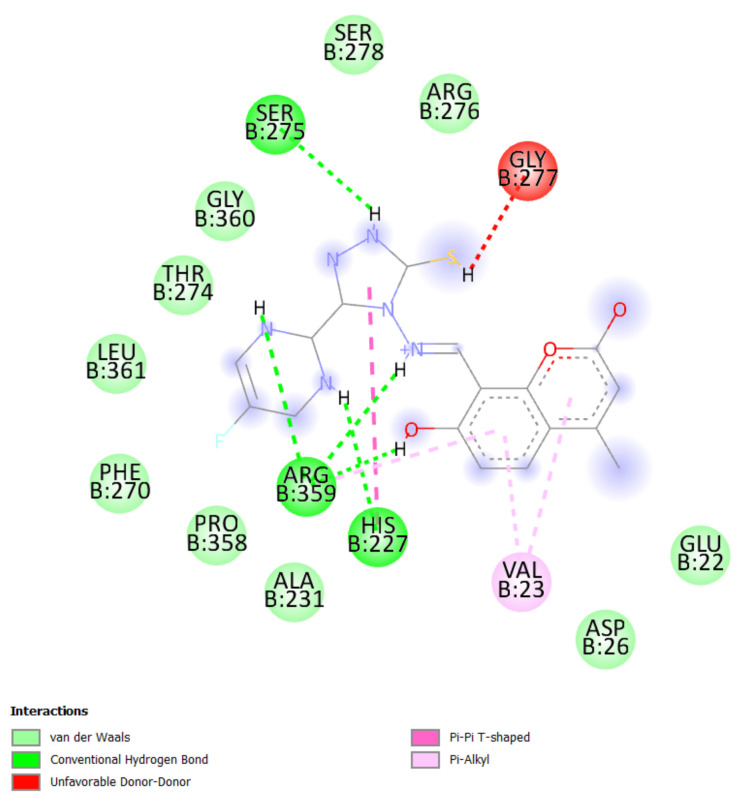
Docking interactions of designed compound **b** with vinblastine binding site of tubulin enzyme of human.

**Figure 11:**
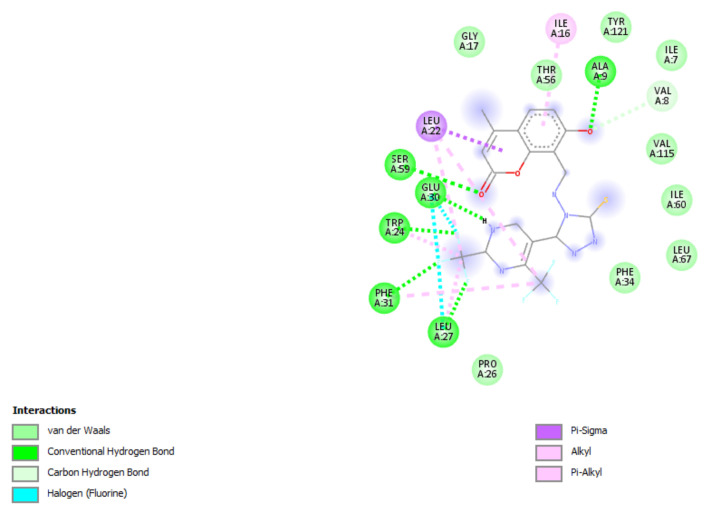
Docking interactions of the designed compound **h** with DHFR enzyme of human.

A molecular docking study was performed in order to study possible interactions between the protein complex and the ligand for the designed compounds. In the present manuscript, we attempted to study the interactions of novel pyrimidine-coumarin-triazole hybrids with DHFR. In order to study the binding efficacy of all the designed compounds **a**–**l**, molecular docking studies were performed in the binding pockets of *S. aureus* dihydrofolate reductase [PDB ID:3SRQ]. All the designed compounds exhibited better binding scores ranging from −6.9 to −9.3 kcal/mol. Binding energies of compounds which target colchicine binding site (PDB ID 1SA0) and vinblastine binding site of tubulin (PDB ID 5J2T) and *Staphylococcus aureus* dihydrofolate reductase [PDB ID: 3SRQ] are presented in Table 6. Docking interactions of the best active compounds are shown in Figure 5, Figure 6, Figure 7, Figure 8 and Figure 9 whereas Figure 10 and Figure 11 represents the binding interactions of designed compounds **b** and **h** on the vinblastine binding site of tubulin and DHFR of human proteins. 

### 2.3. Effects of Predicted Compounds on Nuclear Signaling Pathways were Predicted Using ProTox-II

The compounds were found to be active against three nuclear signaling pathways, namely aromatase, estrogen receptor alpha and estrogen receptor ligand-binding domain (Table 7).

### 2.4. In Silico Studies Using SwissADME Pathways

The prediction of key physicochemical, pharmacokinetic, drug-like and related parameters for one or multiple molecules can be performed by SwissADME. Thus, the SwissADME studies for the designed compounds were carried out (Table 8).

## 3. Discussion

In the present study, a series of compounds reported by Mohit Sandhuja et al. were taken for QSAR analysis. In QSAR model 1 MAXDP is a dimensionless maximal electrotopological positive variation, which correlates the molecule’s electrophilicity and is a measure of the electronic distribution in the topological graph. MAXDP contributes positively to the activity. In previous research, also MAXDP contributed positively to the anticancer activity of coumarin analogs [35]. Information indices are best associated with cytotoxic activity [36]. A positive contribution to the activity was observed for structural information content index (neighborhood symmetry of 0-order) contributes JGI7. The topological charge parameter also contributes positively to the activity. Since two outliers (A2 and A5) were observed in William’s plot, they were removed and QSAR models were again generated. In model 2, AATS7s, MATS2c and SpMin3_Bhi contributed negatively to anticancer activity while MDEC-33 contributed positively. Similar values were found for Q^2^ F1, Q^2^F2 and Q^2^F3, along with elevated CCC (concordance correlation coefficient) parameter values (Figure 1). The results clearly indicate that the best model obtained was not by chance and truly there is a relationship between structures of pyrimidine-coumarin-triazole based trifunctional molecular hybrid analogs with corresponding MCF 7 cell line inhibitory activity.

In model 3, no outliers in William’s plot were observed. The scatter plot of the experimental versus the calculated MCF7 cell line inhibitory activities of pyrimidine-coumarin-triazole based trifunctional molecular hybrids is shown in Figure 1; it shows that predicted values are similar to corresponding experimental values. Figure 2 describes the correlation between the resulting Kxy (the inter-correlation among descriptors and response) versus Q2 LMO of the final model, which shows the LMO parameter values were around the model parameters, meaning the model is robust and stable.

IC1 parameter was newly included in model 4 and it had a negative contribution to the activity. The applicability domain of the model was explained by William’s plot, standardized residuals versus leverage values shown in Figure 4, and it illustrates the prediction and expression. William’s plot indicated that all the molecules are located in the applicability domain of the model with leverage values lower than the warning h* of 0.750. 

All four models showed good statistical values for the training group with R^2^ values greater than 0.9 (equation 1, R^2^ = 0.9700; equation 2, R^2^ = 0.9883; equation 3, R^2^ = 0.9875; equation 4, R^2^ = 0.9874). The cross-validated Q^2^ must be higher for the models to be statistically significant. In all four models Q^2^ value was more than 0.9 (equation 1, Q^2^ = 0.9495; equation 2, Q^2^ = 0.9796; equation 3, Q^2^ = 0.9789; equation 4, Q^2^ = 0.9777,). The difference between R^2^ and Q^2^ should not be more than 0.3. In all four generated models the difference between R^2^ and Q^2^ was found to be 0.009. All of these parameters suggest that the generated QSAR equations have good predictive power. The predicted activities calculated from the best model were found to be close to observed activities.

Twelve new compounds were designed using ChemSketch software. The novelty of the compounds was confirmed by Scifinder. Physicochemical properties of the designed compounds were predicted using the PaDEL descriptor. Their anticancer activity against MCF7 cells was predicted using the generated QSAR models. 

The novel designed compounds exhibited very high inhibitory activity compared to the most active compound of the original data set. The most active compound in the original data set had an IC_50_ value of 1.55. Its pIC_50_ value was found to be 5.809. All the predicted compounds exhibited pIC_50_ values of more than 9.0 except one compound which had a pIC_50_ value of 8.007. The predicted activities against MCF7 cells using the generated QSAR equation for compounds g, h, i and j were found to be 7.9, 7.80, 8.59 and 8.33, respectively. Hence, it was proven that all the predicted compounds are found to be more active against MCF7 cells and could serve as lead compounds for treating breast cancer.

To study the binding efficacy of all the designed compounds **a–l**, molecular docking studies were performed in the binding pockets of *S. aureus* dihydrofolate reductase [PDB ID:3SRQ]. All the designed compounds exhibited better binding scores, ranging from −6.9 to −9.3 kcal/mol. A dock score with a high negative value represents minimum binding energy for the formation of the complex between protein and ligand. The docking study reveals that introduction of an electron-withdrawing group at the second and fourth positions of the pyrimidine ring had a better affinity of the ligand to the protein DHF reductase complex. These compounds exhibited H bond interaction with Ile X-51 and Leu X-21. The Ile formed an H bond with NH of triazole while Leu formed an H bond with the trifluoromethyl group of the pyrimidine nucleus. The two trifluoromethyl groups also formed halogen bonds with Trp X-23, Leu X-21 and Ser X-50. Phe X-93 forms pi-pi stackings with the coumarin nucleus. Ala X-8, Val X-32, Leu X-21, IsoLeu X-51 and Lys 46 are bonded through alkyl interaction. Gly X-20 and His X-24 are linked with the nucleus through van der Waals attraction. Compounds **g**, **h**, **i** and **j** showed excellent binding energies. Their binding energy values were found to be −8.7, −9.0, −9.1 and −8.6, respectively. The binding affinity of novel compounds towards colchicine binding sites of tubulin were in the range of −7.4 to −9.8. The designed compound k showed the highest affinity with a binding value of −9.8. 

Conventional hydrogen bonds are formed between designed compound **k** and Leu-248, Gly-179, Asn-143 and Gln-11 groups of enzymes at distances of 5.40, 4.47, 4.47 and 4.54 angstroms, respectively. Three halogen bonds were formed between the trifluoromethyl group of compounds and Ala-247 at a distance of 5.43 Å. Pi-sigma bonds and pi-pi stacked bonds were formed between the pyrone nuclei of Cys-12 and Gln-11 at distances of 4.80 and 6.95 Å, respectively. Pi anion bonds were formed 6.95 Å away from the triazole fragments of nuclei with Glu-254 of enzymes (Figure 8).

The binding affinity of novel compounds towards vinblastine binding sites of tubulin were in the range of −9.2 to −6. The designed compound **b** showed a binding energy of −9.2. The designed compound b binds with the target by forming two conventional hydrogen bonds with Gln-394 and Val-181 at distances of 4.42 and 2.56 Å, respectively. Two pi-alkyl bonds were formed between the coumarin nucleus and Pro-175 of the target at 5.37 Å. (Figure 9). On comparing the binding affinities between the colchicine binding site and vinblastine binding site of tubulin, it was found that compound **k** with 4-cyclopropyl-2-(trifluoromethyl)pyrimidinyl substitution showed binding affinity towards the colchicine binding site, while compound **b** with 5-fluoropyrimidinyl substitution showed binding affinity towards the vinblastine binding site of tubulin. The interactions of the compounds **g**, **h**, and **i** with the DHFR for protein are presented in Figure 5, Figure 6 and Figure 7.

By comparing the different forms of bond interactions between the protein and ligand among both tubulin targets, interatomic lengths for hydrogen bonds were found to be 2.56 and 5.40 Å, respectively. The hydrogen bonds in both the high- and low-resolution targets of tubulin were found to be at a distance of 4.4 Å. In addition, pi-interaction was identified in both tubulin proteins at a distance of 4.8 to 6.9 Å. Despite the fact that proteins 5J2T and 1SA0 have resolutions of 2.2 and 3.58 Å, respectively, the docking of the ligands showed the same distance range. This illustrates the ligand’s capability of binding to targets of various resolutions. The docking studies were carried out with human tubulin (e.g.,PDB id 6O5N) and DHFR (4KD7) for a comparative study. The results were quite surprising. The amino acid sequence was totally different for human tubulin and rat tubulin. Although the amino acid sequence is different between human and rat, the binding energy of designed compound **b** was found to be −9.0 kcal/mol for human tubulin and −9.4 kcal/mol for rat tubulin (Figure 10). The binding energies for the designed compounds were similar in human tubulin and rat tubulin. In addition, the docking with human DHFR enzyme PDB ID 4KD7 was also carried out for the compounds that exhibited best in the rat DHFR enzyme (PDB ID: 3SRQ). The compounds **g**, **h** and **i** exhibited good binding energies of −9.0, −10.1 and −8.9 kcal/mol, respectively, for human DHFR (Figure 11). The binding sites of the compounds **g**, **h** and **i** vary in both the rat and the human DHFR enzymes, but they bind effectively through hydrogen, van der Waals and pi-stacking interactions in both. By this comparison, we can conclude that the best compounds b and h can serve as the lead for anti-cancer activity in both rat and human breast cells.

The compounds were found to be active against three nuclear signaling pathways, namely aromatase, estrogen receptor alpha and estrogen receptor ligand-binding domain, which was determined by ProTox-II. Aromatase inhibitors work by inhibiting the enzyme aromatase. Aromatase converts the hormone androgen into small amounts of estrogen in the body. Thus, aromatase inhibitors reduce estrogen levels that stimulate the growth of hormone-receptor-positive breast cancer cells [37]. The effects of estrogen are largely mediated by estrogen receptors ER-α and ER-β, which are members of the nuclear receptor superfamily of transcription factors. Estrogen receptor alpha (ER-α) is expressed in approximately 65% of breast cancer cases [38]. Estrogen receptor α is mainly responsible for breast cancer initiation and progression. Since the predicted compounds act as ligands that selectively bind to the estrogen alpha receptor and inhibit estrogen-dependent proliferative activity, they are expected to show anticancer activity. The characterization of estrogen provided a molecular basis for the regulation of estrogen receptors and, thereby a basis to describe the mechanism of the hormone therapy in treating breast cancer [39]. Tamoxifen, a well-known anticancer agent, interferes with all three pathways in ProTox-II, similarly to the designed compounds. Tamoxifen is a stilbenoid. The designed compounds contain coumarin. In general, coumarins are biosynthesised from coumaric acid, which is also a stilbenoid. Thus, coumarins are structurally related to tamoxifen and both of them interfere with aromatase, estrogen receptor alpha and estrogen binding domain.

By the above facts, it was very clear that the predicted compounds could act as lead compounds for breast cancer treatment, and that they can act through all three nuclear signaling pathways. The prediction of key physicochemical, pharmacokinetic, drug-like and related parameters for one or multiple molecules can be performed by SwissADME. Thus, SwissADME studies for the designed compounds were carried out. Qualitative estimation of the class of solubility was conducted according to the following log S scale: insoluble < −10 < poorly < −6 < moderately < −4 < soluble < −2 < very < 0 < highly. The log S scale of the predicted compounds was found to be in the range of −6 to −2. Thus, the designed compounds were found to be moderately soluble to very soluble in water. The compounds were not permeable to the blood-brain barrier. This indicates that they were devoid of CNS side effects.

Cytochrome P_450_1A2 (CYP1A2) is a key enzyme in the cause of breast cancer (BC). It plays a role in activation of breast carcinogen, in the production of beneficial estrogen [2-hydroxyestrone (2-OHE1)] and in converting arachidonic acid (AAc) to epoxyeicosatrienoic acids (EETs), which have anti-inflammatory properties. All the designed compounds were inhibitors of CYP1A2 which would further enhance their anticancer activity [40]. CYP3A4 causes the oxidation of compounds that are usually used as chemotherapeutic agents for the treatment of osteosarcomas such as etoposide, ifosfamide, cyclophosphamide and doxorubicin, suggesting that the response to these drugs could be worse in tumors with high CYP3A expression, increasing the risk of metastasis. All the designed compounds were inhibitors of CYP1A2 which would further enhance the anticancer activities of other drugs, such as etoposide [41]. The synthetic accessibility (SA) score suggests to us the ease of synthesis. The score can be between 1 and 10, where 1 is very easy, while 10 denotes difficulty in synthesizing. The predicted compounds had scores of 3.16–3.71. Thus, these compounds can be synthesized easily. A bioavailability score of 0.55 or 0.56 means a compounds has good pharmacokinetic properties. All the designed compounds showed values of more than 0.55.

The overall results of the above research indicated that the designed compounds exhibited promising results in MCF 7 cell inhibition prediction. The ProTox-II results further confirmed their anticancer activity. The SwissADME results also indicated that the designed compounds are inhibitors of two important enzymes, CYP1A2 and CYP3A4, and thus are predicted to possess anticancer activity.

## 4. Materials and Methods

Multiple linear regression models by ordinary least squares were developed by “QSARINS”, carefully verified and validated in detail according to the chemometric approach. A series containing 28 compounds (Table 1) of uracil–coumarin based bifunctional molecular hybrids linked by 1,2,3-triazole moiety with MCF7 inhibitory values were selected from reported literature. pKi was calculated from observed Ki values and considered as the dependent variable. 

### 4.1. Molecule Structure Preparation and 3D Geometry Optimization

The molecular structures were drawn by ACD/labs ChemSketch freeware 2017.2.116 and converted to mol2 format. Geometry optimization was performed by Avogadro V1.2.018 on adding hydrogens. MMFF94, Merck molecular force field, was employed, along with the steepest descent algorithm. The best conformer with global minimum energy was used throughout the study.

### 4.2. Data Setup

The molecular descriptor values for the compounds were calculated from PaDEL descriptor. All zero values, missing values and constant value (>50%) descriptors were excluded from variables. Descriptors with values greater than 0.85 were filtered out using pair-wise correlation. All twenty-eight compounds were divided into training set and prediction set in a 5:1 ratio. Many trials and models were developed, a few best models are presented in this manuscript.

### 4.3. Variable Selection and Model Calculation

QSARINS software considers all combinations of selected descriptors defined by user options. Descriptor selection relational to biological activities of molecules and Friedman’s “lack-of-fit” (LOF) function was calculated by genetic algorithm. LOF smoothness level is kept at the default level of 1.0. Along with the genetic algorithm, more combinations and maximum generations (user-defined value:2000) were explored by parameters including mutation probability (0.1), population size (200).

### 4.4. Model Validation

Internal validation and external validation, in addition to applicability domain of the model, were performed. Internal validation was performed by cross-validation leave-one-out (Q^2^LOO), cross-validation leave-many-out (Q^2^LMO), root mean squared error (RMSE), Y-scrambling, and external validation by Q^2^ F1, Q^2^ F2, and Q^2^ F3,22; CCC was applied on selected models. Q^2^LMO was repeated 2000 times with 30% of objects left from the training set each time. Y-scrambling was performed by 2000 iterations method in order to exclude chance correlation in the original model. R^2^ and Q^2^LOO of the model must be reasonably higher than scrambled ones, and RMSE of the model underprediction must be reasonably smaller than scrambled ones. The concordance correlation coefficient (CCCext) was analyzed. The leverage (hat) was calculated by hi = xi (XT X)-1 xTi (I = 1, 2, …m), where xi is the descriptor row-value of the query compound i, and m is the number of query compounds. X is n × *p* matrix of the training set, where n is the number of training set samples and *p* is the number of model descriptors. The leverage cut-off value h* is 3(*p* + 1)/n. Leverage greater than h* for the training set means that the sample is highly influential in determining the model, whereas in the test set (X outlier) the prediction is extrapolation of the model. Any compound with a standardized residual of more than 3σ (3 standard deviation units) is identified as a Y outlier.

### 4.5. Molecular Docking Study

Molecular docking protocols are widely used for predicting the binding affinities for a number of ligands. Intermediary steps, such as PDBQT files for protein and ligands preparation and grid box creation, were completed using the graphical user interface program AutoDock Tools (ADT). ADT assigned polar hydrogens, united atom Kollman charges, solvation parameters and fragmental volumes to the protein. AutoDock saved the prepared file in PDBQT format. AutoGrid was used for the preparation of the grid map using a grid box. The grid size was set to 60 × 60 × 60 xyz points with a grid spacing of 0.375 Å and grid center was designated at dimensions (x, y and z): −1.095, −1.554 and 3.894. A scoring grid is calculated from the ligand structure in order to minimize computation time. AutoDock Vina was employed for docking using protein and ligand information along with grid box properties in the configuration file. AutoDock Vina employs iterated local search global optimizer. During the docking procedure, both the protein and ligands are considered as rigid. Results less than 1.0 Å in positional root-mean-square deviation (RMSD) were clustered together and represented by the result with the most favorable free energy of binding. The pose with the lowest energy of binding or binding affinity was extracted and aligned with receptor structure for further analysis.

Hydrogen bonds and Gasteiger–Huckel charges were assigned to the protein of interest and designed compounds using Chimera software. The cofactors and water molecules were also eliminated from the protein. A molecular docking study was performed using PyRxAutodock Vina in the binding site of *Staphylococcus aureus* dihydrofolate reductase [PDB ID:3SRQ]. The grid box of dimensions in Å were: center (X, Y, Z) = (−2.5, 0.32, −21.67), dimensions (X, Y, Z) = (88.31, 88.31, 88.31), with an exhaustiveness of 8. The docking poses for protein–ligand interactions were chosen on the basis of docking scores. The pose with the highest docking score was selected. The binding interactions were developed using the Molegro molecular viewer software.

### 4.6. In Silico Studies

Effect of predicted compounds on nuclear signaling pathways was predicted using ProTox-II. The effect of compounds on aryl hydrocarbon receptor (AhR), androgen receptor (AR), androgen receptor ligand binding domain (AR-LBD), aromatase, estrogen receptor alpha (ER-α), estrogen receptor ligand b inding domain (ER-LBD) and peroxisome proliferator activated receptor gamma (PPAR-Gamma) nuclear signaling pathways were checked. SwissADME Web determines physicochemical, pharmacokinetic, drug-like and related parameters for multiple molecule; thus, SwissADME studies for the designed compounds were carried out.

## 5. Conclusions

In continuation of our research on coumarin derivatives [42,43], we report in this study the 2D-QSAR studies on pyrimidine-tethered coumarin–triazole derivatives. The models developed with 2D descriptors were found to be robust, stable and predictive. A study to discover the role of 2D descriptors in tubulin and dihydrofolate reductase inhibiting activity was carried out by developing a good statistical model. Model validation was performed internally and externally. The model was tested on designed compounds for predicting bioactivity. The best predicted active compounds were subjected to molecular docking studies for structural and interaction information supporting our QSAR model. The compounds **c**, **d**, **h**, **i** and **j** were found to have potential DHFR inhibitory activity and are being considered for further synthesis and biological screening.

## Figures and Tables

**Table 1:**
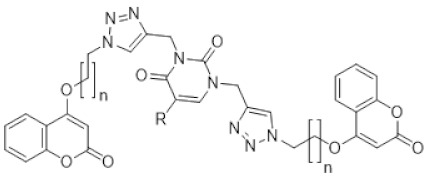
GI_50_ (mM) values of synthesized hybrids.

Code	R	n	pIC_50_
**1**	H	1	10.99
**2**	F	1	1.55
**3**	Cl	1	2.57
**4**	Br	1	3.34
**5**	I	1	3.96
**6**	CH_3_	1	5.16
**7**	NO_2_	1	4.25
**8**	H	2	13.57
**9**	F	2	3.34
**10**	Cl	2	4.23
**11**	Br	2	4.71
**12**	I	2	5.73
**13**	CH_3_	2	9.88
**14**	NO_2_	2	6.41
**15**	H	3	16.63
**16**	F	3	5.15
**17**	Cl	3	5.99
**18**	Br	3	6.88
**19**	I	3	7.75
**20**	CH_3_	3	11.33
**21**	NO_2_	3	9.77
**22**	H	4	19.55
**23**	F	4	6.05
**24**	Cl	4	6.71
**25**	Br	4	7.29
**26**	I	4	9.33
**27**	CH_3_	4	14.75
**28**	NO_2_	4	12.24

**Table 2:** Descriptor correlation matrix for the best model.

	AATS7s	MATS2c	SpMin3_Bhi	MDEC-33
AATS7s	1.000			
MATS2c	−0.6597	1.000		
SpMin3_Bhi	0.2951	0.1291	1.000	
MDEC-33	0.5981	−0.5151	0.3539	1.000

**Table 3:** pKi values of the original data set predicted by the best model equation.

S.No	AATS7s	MATS2c	SpMin3_Bhi	MDEC-33	Observed Activity	Predicted Activity	Residual
1	3.373101	0.041687	1.704602	10.0791391	5.59	5.564453	0.025547
2	3.351496	0.048774	1.70672	10.0791391	5.476	5.522631	−0.04663
3	3.348425	0.062204	1.709487	10.0791391	5.402	5.450572	−0.04857
4	3.357474	0.075511	1.729188	10.0791391	5.287	5.237408	0.049592
5	4.110095	0.03852	1.723253	10.0791391	5.372	5.360054	0.011946
6	2.850694	0.09839	1.701321	7.7983348	4.867	4.870679	−0.00368
7	3.455229	0.044554	1.698517	9.42319018	5.476	5.437684	0.038316
8	2.985512	0.06304	1.705109	9.42319018	5.374	5.354074	0.019926
9	2.965686	0.070285	1.707205	9.42319018	5.27	5.311702	−0.0417
10	2.962868	0.083909	1.709945	9.42319018	5.242	5.23913	0.00287
11	2.996642	0.097217	1.729488	9.42319018	5.005	5.025225	−0.02022
12	3.68196	0.059358	1.723591	9.42319018	5.193	5.156333	0.036667
13	2.867544	0.104709	1.695475	7.32266243	4.779	4.778616	0.000384
14	3.304386	0.043052	1.698914	8.86256391	5.288	5.315756	−0.02776
15	2.884113	0.061294	1.705445	8.86256391	5.222	5.229445	−0.00745
16	2.866374	0.068444	1.707524	8.86256391	5.162	5.187387	−0.02539
17	2.863853	0.081897	1.710244	8.86256391	5.11	5.115595	−0.0056
18	2.900165	0.095095	1.729668	8.86256391	4.946	4.90286	0.04314
19	3.51431	0.057675	1.723799	8.86256391	5.01	5.038041	−0.02804
20	2.794935	0.100075	1.695788	6.91156492	4.709	4.698848	0.010152
21	3.201797	0.03936	1.699197	8.37672004	5.218	5.217054	0.000946
22	2.810367	0.057278	1.705677	8.37672004	5.173	5.12996	0.04304
23	2.793845	0.06431	1.707742	8.37672004	5.137	5.088354	0.048646
24	2.791497	0.077555	1.710444	8.37672004	5.03	5.017453	0.012547
25	2.829475	0.09062	1.729779	8.37672004	4.831	4.80581	0.02519
26	3.40173	0.053783	1.72393	8.37672004	4.912	4.942162	−0.03016
27	3.373101	0.041687	1.704602	10.0791391	5.59	5.564453	0.025547
28	3.351496	0.048774	1.70672	10.0791391	5.476	5.522631	−0.04663
29	3.348425	0.062204	1.709487	10.0791391	5.402	5.450572	−0.04857
30	3.357474	0.075511	1.729188	10.0791391	5.287	5.237408	0.049592
31	4.110095	0.03852	1.723253	10.0791391	5.372	5.360054	0.011946
32	2.850694	0.09839	1.701321	7.7983348	4.867	4.870679	−0.00368
33	3.455229	0.044554	1.698517	9.42319018	5.476	5.437684	0.038316
34	2.985512	0.06304	1.705109	9.42319018	5.374	5.354074	0.019926
35	2.965686	0.070285	1.707205	9.42319018	5.27	5.311702	−0.0417
36	2.962868	0.083909	1.709945	9.42319018	5.242	5.23913	0.00287
37	2.996642	0.097217	1.729488	9.42319018	5.005	5.025225	−0.02022
38	3.68196	0.059358	1.723591	9.42319018	5.193	5.156333	0.036667
39	2.867544	0.104709	1.695475	7.32266243	4.779	4.778616	0.000384
40	3.304386	0.043052	1.698914	8.86256391	5.288	5.315756	−0.02776
41	2.884113	0.061294	1.705445	8.86256391	5.222	5.229445	−0.00745
42	2.866374	0.068444	1.707524	8.86256391	5.162	5.187387	−0.02539
43	2.863853	0.081897	1.710244	8.86256391	5.11	5.115595	−0.0056
44	2.900165	0.095095	1.729668	8.86256391	4.946	4.90286	0.04314
45	3.51431	0.057675	1.723799	8.86256391	5.01	5.038041	−0.02804
46	2.794935	0.100075	1.695788	6.91156492	4.709	4.698848	0.010152
47	3.201797	0.03936	1.699197	8.37672004	5.218	5.217054	0.000946
48	2.810367	0.057278	1.705677	8.37672004	5.173	5.12996	0.04304
49	2.793845	0.06431	1.707742	8.37672004	5.137	5.088354	0.048646
50	2.791497	0.077555	1.710444	8.37672004	5.03	5.017453	0.012547
51	2.829475	0.09062	1.729779	8.37672004	4.831	4.80581	0.02519
52	3.40173	0.053783	1.72393	8.37672004	4.912	4.942162	−0.03016

**Table 4:**
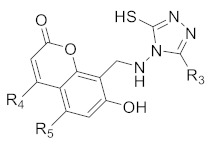
Structures of the predicted compounds **a–l**.

S.NO	Compound Code	Substituents		
		**R_3_**	**R_4_**	**R_5_**
1	**a**	5-fluoropyrimidinyl	-H	-H
2	**b**	5-fluoropyrimidinyl	-CH_3_	-H
3	**c**	5-fluoropyrimidinyl	-H	-OCH_3_
4	**d**	2-(trifluoromethyl)pyrimidinyl	-H	-H
5	**e**	2-(trifluoromethyl)pyrimidinyl	-CH_3_	-H
6	**f**	2-(trifluoromethyl)pyrimidinyl	-H	-OCH_3_
7	**g**	2,4-bis(trifluoromethyl)pyrimidinyl	-H	-H
8	**h**	2,4-bis(trifluoromethyl)pyrimidinyl	-CH_3_	-H
9	**i**	2,4-bis(trifluoromethyl)pyrimidinyl	-H	-OCH_3_
10	**j**	4-cyclopropyl-2-(trifluoromethyl)pyrimidinyl	-H	-H
11	**k**	4-cyclopropyl-2-(trifluoromethyl)pyrimidinyl	-CH_3_	-H
12	**l**	4-cyclopropyl-2-(trifluoromethyl)pyrimidinyl	-H	-OCH_3_

**Table 5:** pKi values of designed compounds predicted by the best model equation.

Compound Code	AATS7s	MATS2c	SpMin3_Bhi	MDEC-33	Predicted Activity
**a**	4.65426	−0.08643	1.422487	8.99956	8.007063
**b**	4.887336	−0.06669	1.422521	13.38021	8.982861
**c**	4.686847	−0.06804	1.473045	15.7691	9.167553
**d**	4.620868	−0.0867	1.422633	15.7691	9.660035
**e**	4.385983	−0.08759	1.472606	11.30879	8.180639
**f**	4.254474	0.042657	1.415929	11.30879	8.18336
**g**	5.166064	−0.07107	1.420907	8.99956	7.921204
**h**	4.912833	−0.01094	1.478859	11.30879	7.803298
**i**	4.755541	−0.09143	1.421056	11.30879	8.591879
**j**	5.677675	−0.05352	1.422787	11.1857	8.331555
**k**	5.329807	−0.05452	1.473214	13.54342	8.520435
**l**	4.755541	−0.09143	1.421056	11.30879	8.591879

**Table 6:** Binding energies of compounds targeting colchicine binding site (PDB ID 1SA0) and vinblastine binding site of tubulin (PDB ID 5J2T) and *S. aureus* dihydrofolate reductase [PDB ID: 3SRQ].

Compound Code	Binding Energy (kcal/mol)		
	Tubulin-Colchicine	Tubulin-Vinblastine	DHFR
**a**	−8.7	−7.9	−7.4
**b**	−9.4	−9.2	−7.7
**c**	−8.8	−8.6	−8.7
**d**	−8.8	−8.5	−6.9
**e**	−8.7	−7.6	−7.4
**f**	−8.9	−8.3	−8.9
**g**	−9.1	−8.7	−8.7
**h**	−8.5	−8	−9.0
**i**	−7.9	−7.1	−9.1
**j**	−9.6	−6.9	−8.6
**k**	−9.8	−6.4	−8.5
**l**	−9	−7.1	−8.4

**Table 7:** Effect of predicted compounds on nuclear signaling pathways predicted using ProTox-II.

S.No	Aryl Hydrocarbon Receptor	Androgen Receptor	Androgen Receptor Ligand Binding Domain	Aromatase	Estrogen Receptor Alpha (ER)	Estrogen Receptor Ligand Binding Domain (ER-LBD)	Peroxisome Proliferator Activated Receptor Gamma (PPAR-Gamma)
1	Inactive	Inactive	Inactive	Active	Active	Active	Inactive
2	Inactive	Inactive	Inactive	Active	Active	Active	Inactive
3	Inactive	Inactive	Inactive	Active	Active	Active	Inactive
4	Inactive	Inactive	Inactive	Active	Active	Active	Inactive
5	Inactive	Inactive	Inactive	Active	Active	Active	Inactive
6	Inactive	Inactive	Inactive	Active	Active	Active	Inactive
7	Inactive	Inactive	Inactive	Active	Active	Active	Inactive
8	Inactive	Inactive	Inactive	Active	Active	Active	Inactive
9	Inactive	Inactive	Inactive	Active	Active	Active	Inactive
10	Inactive	Inactive	Inactive	Active	Active	Active	Inactive
11	Inactive	Inactive	Inactive	Active	Active	Active	Inactive
12	Inactive	Inactive	Inactive	Active	Active	Active	Inactive

**Table 8:** SwissADME results of the predicted compounds.

Compound Number	ESOL Log S	No. of H Bond Acceptors	No. of H Bond Donors	GI Tract Absorption	Lipophilicity Q log P_o/w_	Lipinski Drug- Likeness
**a**	−3.51	8	2	Low	2.55	0 violation
**b**	−3.53	8	2	Low	2.66	0 violation
**c**	−4.2	10	2	Low	1.95	0 violation
**d**	−4.21	10	2	Low	2.17	0 violation
**e**	−3.57	9	2	Low	2.07	0 violation
**f**	−4.26	11	2	Low	2.19	0violation
**g**	−5.07	13	2	Low	2.67	1 violation
**i**	−5.09	13	2	Low	2.5	1 violation
**j**	−5.15	14	2	Low	2.73	1 violation
**k**	−4.69	10	2	Low	3.56	0 violation
**l**	−4.71	10	2	Low	2.91	0 violation
**m**	−4.76	11	2	Low	3.23	1 violation

## Data Availability

Not applicable.
